# Retraction: Design of a tomato classifier based on machine vision

**DOI:** 10.1371/journal.pone.0326683

**Published:** 2025-06-23

**Authors:** The PLOS One Editors

The *PLOS One* Editors retract this article [1] because it was identified as one of a series of submissions for which we have concerns about compromised peer review. We regret that the issues were not identified prior to the article’s publication.

All authors either did not respond directly or could not be reached.
